# Surgery Training System Supported by Organic Materials

**DOI:** 10.3390/ma15124162

**Published:** 2022-06-12

**Authors:** Magdalena Błaszczyk, Jadwiga Gabor, Tomasz Flak, Zygmunt Wróbel, Andrzej S. Swinarew

**Affiliations:** 1 Faculty of Science and Technology, University of Silesia in Katowice, 40-007 Katowice, Poland; magdalena.blaszczyk@us.edu.pl (M.B.); jadwiga.gabor@us.edu.pl (J.G.); tomasz.flak@us.edu.pl (T.F.); zygmunt.wrobel@us.edu.pl (Z.W.); 2 Medicus Sp. z o.o., 50-224 Wrocław, Poland; 3 Institute of Sport Science, The Jerzy Kukuczka Academy of Physical Education, 40-065 Katowice, Poland

**Keywords:** polycarbonate, craniofacial implant, extraoral implant, 3D printing

## Abstract

The aim of the study was the qualitative assessment of new materials based on a polycarbonate matrix in terms of its use in 3D printing and its processing and geometric modification (cutting). Filaments made of the new material doped with talc in five different proportions were visually inspected with a microscope. The calibration and test models were made using the FFF (fused filament fabrication) technique. In addition, its susceptibility to the drill and the behavior of the shavings were assessed and the temperature changing during drilling was measured. The implant was inserted to measure its resonance stability in each of the holes made and translated into the value of the implant stability quotient (ISQ) ranging from 1 to 100. The results were compared to those obtained for the training model of the skull bone. The amount of filler has been shown to affect the composite. Moreover, due to the properties of talc, a compatibilizer (polyol) was used. Differences were observed between the model made of the commercial material, the model made of the dried, tested material, and the model made of the undried material. It was confirmed that the presence of water in the material during its processing is important.

## 1. Introduction

In recent years, medicine has paid great attention to holistic, comprehensive treatment, taking into account the improvement of the quality of life of an oncological patient. Such an approach positively influences the patient’s recovery and their reactivation in society.

A special case is extensive craniofacial resection, where neoplastic lesions are removed with a large margin of tissue or the tumor itself is so extensive that the patient is deprived of a large part of the face (e.g., nose, eyeball with adjacent structures, or auricle). A different appearance is so stigmatizing that patients often give up social life, retire professionally, and fall into depression. Therefore, extensive facial defects almost always lead to a high emotional burden requiring rehabilitation [1].

In many cases, the cavities are so extensive that the use of classic solutions, such as retention dentures or plastic surgery, is impossible, or the aesthetic effect may be disappointing [2,3]. In such cases, an opportunity may be a bone-anchored prosthesis system, which belongs to the group of solitary implants due to the attachment method. This group is characterized by the fact that one or several implants form an independent anchoring point for epithesis in the bone (primary stability—obtained by threading titanium in the bone and biological stability, i.e., osseointegration—titanium possesses high biocompatibility in bones [4,5] owing to which, from a biological perspective, it allows for achieving optimal fixation and maintaining it in the bone during functional implant loading [6]).

The qualitative and visual effect of bone-anchored prostheses is very good [7], and their easy and repeatable placement in the correct position without the need for adhesives contributes to the comfort of wearing them and their slower wear [8].

Despite many years of experience with craniofacial implants, some surgical challenges and difficulties make it impossible to clearly predict the final anchoring effect of facial epithesis. A set of factors affect osseointegration [9]. Those are:

- The material of the fixture to be implanted (implant VXI300 is commercially pure grade 1 unalloyed titanium (ASTM standard F-67) [10]);
- The macrostructure of the implant (the self-tapping screws with a characteristic screw-thread [5,11]);
- The microstructure (implant covered with titanium oxide: TiO blast structure);
- The bone bed where the implant is anchored, the surgical non-traumatic technique, and the load of the implant (preferably in the longitudinal direction);
- The surgical technique (following an appropriate surgical procedure, including preventing bone overheating and avoiding applying biofilm to the implant [12]);
- The implant load (implant diameter, i.e., the contact surface of the implant with the bone [3]).

Since the beginning of the existence of bone-anchored prostheses, the concept of stability has been extremely important and is directly related to the maintenance of implants in the patient’s body, and thus determines the success of the patient’s treatment and rehabilitation. The main factors of importance to lose osseointegration are: minor torsion forces, quality of bone, bone that has been irradiated, and chemotherapy [9]. Oncological patients after radiation therapy belong to the most difficult group because the bone quality in the irradiated area decreases to a great extent and the site is not easily accessible [13,14,15,16]. In addition, it can be changed by tumour resection procedures or further treatment [3]. The possibility of additional planning and practical training of implant placement before the planned procedure may be helpful and give a chance to increase the success of implantation.

A key step for the successful anchoring of the implants is planning the place of their placement. Considering the many variables; anatomical issues; and other risks, the precision of implantation is extremely important and directly related to maintaining stability; support; and behavior, and to avoiding placement of implants that would negatively impact the aesthetics of the contours of the prosthesis. Computed tomography (CT) or magnetic resonance imaging (MRI) is helpful, but it is usually difficult to map the implantation sites and visualize them without first trying on a training model.

Preparing the VXI300 implant holes from Cochlear (Mölnlycke, Sweden), and then anchoring the implant as a medical procedure is detailed in the Surgical Manual Ref. E82283 and E82083A from the manufacturer [12]. For training purposes, the skull model, plastic temporal bone, ref. 90644, is manufactured from a commercial material by casting. The choice of material used for surgical training will be important. In the context of work comfort and facilitating the drilling process, a material with a greater roughness would limit the sliding of the drill on the model. The minimization of inhomogeneity features (e.g., air bubbles) and the degree of its brittleness would also have a positive effect and reduce the degree of dusting and electrification of chips during cutting. In the context of the training surgical procedure and the increase in training values, it is worth paying attention to the fact that the model used for this purpose should have parameters as similar as possible to the bone tissue, including thermal properties where the temperature of the working drill would rise slowly. Moreover, in this case, an advantage is a material that is not susceptible to UV radiation but suitable for sterilization or disinfection.

The above remarks are an inspiration for research aimed at proposing a new material, from which it will be possible to create a training model for use in preoperative or surgical conditions facilitating the implantation planning process.

More and more often 3D printing is used in medicine, completely changing the healthcare system. It enables the creation of visual and training models for planning and simulating surgical procedures [17], as well as personalized treatment through individually tailored implants, prostheses, building biomedical models and adapted surgical tools, as well as bioprinting tissues and living scaffolds for regenerative medicine [18,19]. One of the methods of 3D printing is fused deposition modeling (FDM) or fused filament fabrication (FFF), which is commonly used due to its capabilities when it comes to printing elements as well as low costs [18].

Nowadays, using new technologies in planning the treatment of craniofacial defects and preoperative planning is extremely important. New materials with properties similar to the bone and 3D printing will be key here. Moreover, such a material should be characterized by specific mechanical parameters (flexibility, brittleness, strength), thermal (resistance to high temperatures—sterilization, cutting), biological (antibacterial, biocompatibility), economic aspects (ease of manufacture, cost). Ultimately, the material should be able to be used for medical purposes.

Polycarbonate (PC) appears to meet the above requirements. It is widely used and has strength and stiffness in the vicinity of bone tissue [20]. It does not adversely affect cell proliferation [19]. It is plastic, and thanks to thermal treatment, it can take the desired shape. It is possible to modify it with powder fillers not only to improve mechanical properties, but also to obtain an antibacterial surface [21].

It was decided to use PC as a matrix material to create a bone simulating composite. According to the material sheet for Covestro Macrolon 2600 Polycarbonate [22], it is characterized by hardness and compressive strength (modulus of elasticity 2.35 GPa), similar to aluminum. It is advisable to increase material brittleness, which affects the composite’s mechanical properties and impact strength, to obtain a material resembling human skull bone (modulus of elasticity 10.40–19.60 GPa [23]).

When designing the composite, one should also pay attention to the differences in PC density of 1.20 g/cm^3^, which is below the range of skull bone density (1.68–1.87 g/cm^3^ [23]). Polycarbonate is resistant to high temperatures (processing temperature from 280 °C to 310 °C), which allows for a wide range of use (at temperatures from −100 °C to 135 °C). It enables steam sterilization using an autoclave, where the process temperature reaches 134 °C, as well as gamma radiation or ethylene oxide [24]. Thanks to the resistance to UV radiation, the material can also be sterilized by ultraviolet germicidal irradiation (UVGI) method. However, some studies indicate the phenomenon of material aging under the influence of ultraviolet radiation [25,26], where attention is drawn to the deterioration of mechanical properties of PC.

Moreover, the material has a low creep tendency, which translates into the accuracy of reproducing the printed models, even many months after their printing (it may be useful during multi-stage surgery planning).

Due to their structure, polycarbonates—linear saturated polyesters of carbonic acid and dill—can change their chemical structure under the influence of moisture and become more brittle. This is one of the few disadvantages but very important because it directly impacts the final results of the processing process. This is because the polymer is hydrolyzed at temperatures above 240 °C. In order to eliminate the negative influence of water during 3D printing with the fused filament fabrication or fused deposition modeling technique, where the nozzle temperature reaches 295 °C, the material is dried before the process (3–4 h at 100–120 °C) to achieve the permissible content moisture <0.05% [27] and even 0.015% [28]. It is very important, according to the material card, water absorption at saturation is 0.30% for thickness 0.100 mm (for ISO 62) and water vapor transmission 15.0 g/m^2^/day (for ISO 15106-1) [22].

To improve the properties of the material, additives are used, e.g., in the form of talc. Talc is a mineral from the silicate family. This material was chosen for its properties—it is characterized by very good solubility in polymers. In addition, it has an antistatic and anti-adhesive effect. During the processing of the printed models (drilling), it may positively affect the comfort of the person making the holes.

The size of the talc particles is 3–9 μm, however, due to its lamellar structure, where the two SiO_4_ planes are separated by Mg(OH)_2_, Van der Waals forces occur at the molecular level. The lamellar structure also reduces the shrinkage effect of the polymer. In addition, although a small processing shrinkage characterizes PC itself at the level of 0.6% to 0.8%, the addition of talc additionally improves the dimensional stability [29], which will be beneficial for the created composite models.

The advantage of talc in terms of use in medicine is also its hydrophobicity and chemical inertness.

In addition to the admixture and matrix material, a compatibilizing agent is used to produce the composite. The use of an inorganic admixture (talc) in a matrix made of a polymeric material (polycarbonate) could lead to an inhomogeneous distribution of the admixture particles in the material because they are extremely different from one another in terms of their chemical structure and structure, which would result in the formation of low-energy talc agglomerates. In turn, the formation of large clusters and precipitations of talc would give rise to significant inhomogeneities, which would result in large isotropy of properties. Therefore, in order to avoid it, the admixture must be evenly distributed throughout the entire volume of the material, and, therefore, we use a compatibilizing agent—polyol (PO), which is very compatible with polycarbonate and at the same time inorganic impurities, e.g., talc, are very well suspended in it. The use of polyol results in an even distribution of talcum admixture particles in the PC without any agglomerates with low internal energy.

Increasing the interaction between the matrix and the filler significantly impacts the cohesiveness and homogeneity of the material and, consequently, its processing and strength properties [26,30,31]. 

The study aimed to create a material suitable for processing by 3D printing, which will allow the possibility of making a personalized surgical training model.

## 2. Materials and Methods

### 2.1. Composite

The material is created based on the methodology contained in the patent. PL 227,529 [32]. To create the composite, PC (Covestro Makrolon 2600 Polycarbonate, Songhan Plastic Technology, Shanghai, China) and a compatibilizer from the group of polyhydric alcohols—polyol (poly (propylene oxide) Rokpol D2002, PCC Rokita, Brzeg Dolny, Poland) were used. The polyol acted as a particle stabilizer as well, as it allowed a reduce in talc dusting during the homogenization process: mixing 20–300 rpm for 30 min (CAT R50 homogenizer, Merazet, Poznań, Poland). Before further use, the PC was subjected to a drying process using the ChemLand DZ-3BCII dryer (Chemland, Krakow, Poland) for 24 h at a temperature of 100 °C.

A screw extruder was used in the extrusion process. Prepared mixtures were separately fed into the hopper of a five-zone single screw extruder L/D = 25 PLV 151 (Brabender, Duisburg, Germany,) and formed into a string using a nozzle. In order to plasticize the material, the following temperatures were used in individual zones: I*—32 °C, II—240 °C, III—255 °C, IV—265 °C, V**—265 °C (*—hopper; **—extrusion head). The initial temperature range was determined by data specific for pure PC and was refined during extrusion based on an empirical evaluation of the parameters of the process, taking into account the extruder operating pressure and the extrudate acquisition rate. Filaments with a diameter of 1.75 mm were made from a new material based on talc-doped polycarbonate with the composition shown in the table below (Table 1). The proposed composition was selected experimentally based on the experience gained during the creation of the patent PL 228,980 [33]. The jump of talc content was selected to show changes in the properties of the produced composite. Among other things, the key factor is the fact that the migration of the admixture to the material surface is significantly limited due to the presence of PO particles characterized by a star-shaped shape and a high molecular weight [33].

Optical microscopy (Carl ZEISS Vario700 microscope (Carl Zeiss, Oberkochen, Germany), magnification 19×, was used in the qualitative analysis of the produced filaments. The device allowed us to determine the structure of the material. Due to the transparency of the material, it was also possible to determine the interior of the filament structure.

### 2.2. 3D Printing and Visual Assessment of the Printing Effect

**Figure 1:**
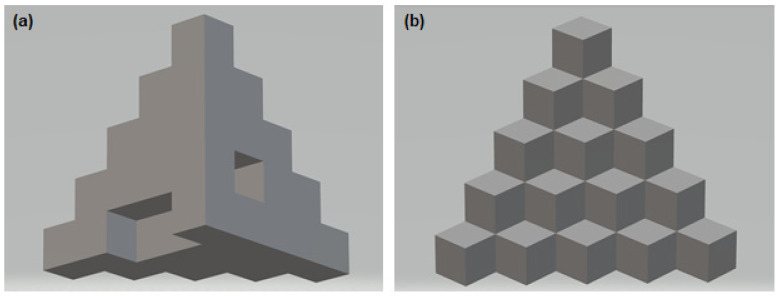
Three-dimensional calibration models (**a**) back, (**b**) front with the following dimensions: object overall x, y, z dimension = 25 mm. Each layer of cubes is 5 mm high. Base layer cutout: 5 mm high, 5 mm deep, 10 mm wide. The 2nd layer cutout: 5 mm high, 5 mm deep, 5 mm wide.

**Figure 2:**
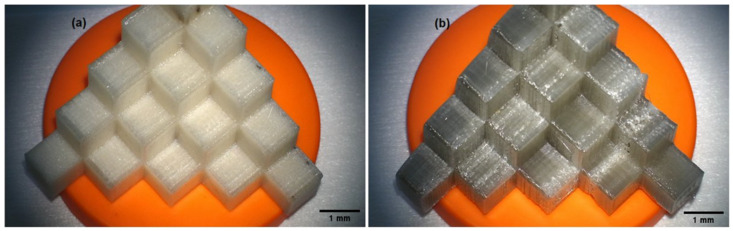
Calibration models printed on the VORON model 2.4 printer. Filament printout: (**a**) material in the test model with the composition of 700 g PC/5 g PO/3.5 g talc and (**b**) material in the test model with the composition 700 g PC/5 g PO/3.5 g talc, additionally free of water using the ChemLand DZ dryer -3BCII.

Using a 3D printer (VORON model 2.4.) based on the standardized 3D model, available under the number 24,238 (Figure 1) [34], three-dimensional calibration and test models (Figure 2) were produced. 

The printing temperature on the head was set at 295 °C, and the bed temperature was set at 126 °C for the first layer, kept at 116 °C. The maximum linear printing speed was 120 mm/s.

The model’s original shape was used for the conducted research—each step was a place for a single borehole and implant insertion. 

The shape of the model was selected to fit the assumptions of this study. Each area needed to be independent for comparing temperature parameters and implant stability. At the same time, the homogeneous shape of the solid with a wide base guaranteed the stability of the model during drilling, and its size made it possible to lock the model in a vice optionally. As a result, drilling at separate levels was important to reproduce the results and allowed for the reproduction of the method of inserting the drill or implant. It made it possible to ignore the influence of adjacent areas (such as temperature increase due to local overheating of the material, too small distance between the boreholes).

The dimensions of the original model of the object were changed to correspond to the proportions of the drills and implants used. As a result, the size of each of the 15 cubes was 10 mm high, 10 mm wide, and 10 mm deep. The object prepared in this way was printed on a printer with the use of produced filaments.

### 2.3. Machining, Temperature Measurement and Evaluation of the Implant Stability

**Figure 3:**
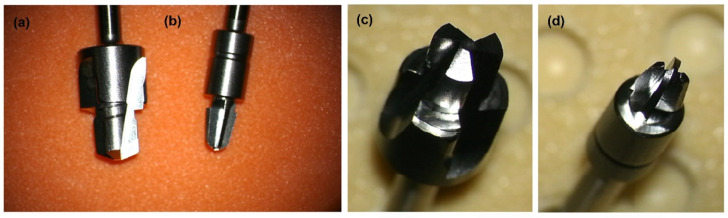
(**a**,**c**) Widening drill (depth 4 mm and diameter 4.1 mm) with countersink (depth 0.5 mm and diameter 5.5 mm). (**b**,**d**) Conical guide drill (depth 3 + 4 mm and diameter 3.1 mm), which is described in detail in patent [35]. All drills are made of hardened stainless steel (WS 1.4197). Pictures taken with the Carl ZEISS Vario700 microscope, 6.5× magnification; focal length 225–235 mm.

The changing properties of the material were then investigated by drilling with a hardened stainless steel drill (Cochlear, Mölnlycke, Sweden) at a speed of 2000 rpm using an Osscora surgical kit also from Cochlear. The metal alloy, shape, and dimensions of the drills are shown below (Figure 3).

In addition to the qualitative assessment of the material produced, its susceptibility to the drill, and the behavior of shavings, the temperature changing during drilling were measured, and the implant was inserted to measure its stability in each of the holes made.

**Figure 4:**
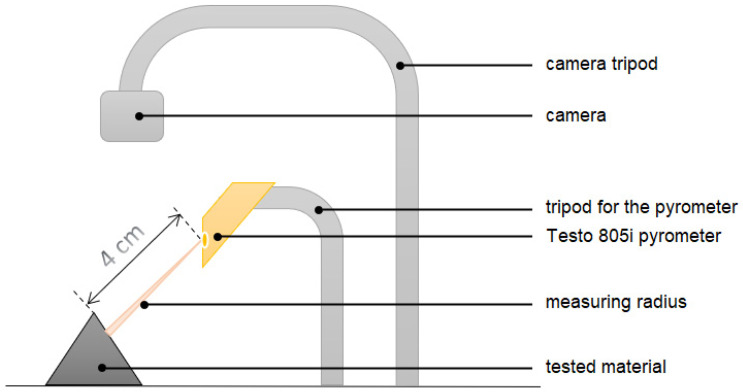
Non-contact measurement of temperature change during cutting-measuring system. A Testo 805i pyrometer (Pruszków, Poland) measured the temperature. Constant room temperature 21.6 °C, air-conditioned room. At a distance of 4 cm, the value of the temperature reading averaged over the area of 0.4 cm diameter area.

The temperature change during the processing of the produced model can be determined by measuring its local increase at the contact point of the titanium drill and the edge of the material in which the hole was made (according to the measurement scheme of Figure 4). For this purpose, a testo 805i pyrometer (Pruszków, Poland) accuracy ± 1.5% of the measured value (0 to +250 °C) was used. The temperature around the drill hole was measured right after the drill was removed with a pyrometer set at a distance of 4 cm (optics 10:1). The accuracy of the result was maintained by the diffraction lens as laser marking (laser circle), which facilitated the determination of the location of the measurement point from which the temperature was read.

Using a caliper, the geometrical dimensions of the holes were characterized (diameter, depth). Dimensions determine the success of introducing the VXI300 implant (Cochlear, Mölnlycke, Sweden), and thus affect possible stresses, bone defects, or inflammation that may appear around the implant, which may ultimately affect the final stability of the Vistafix system [36].

**Figure 5:**
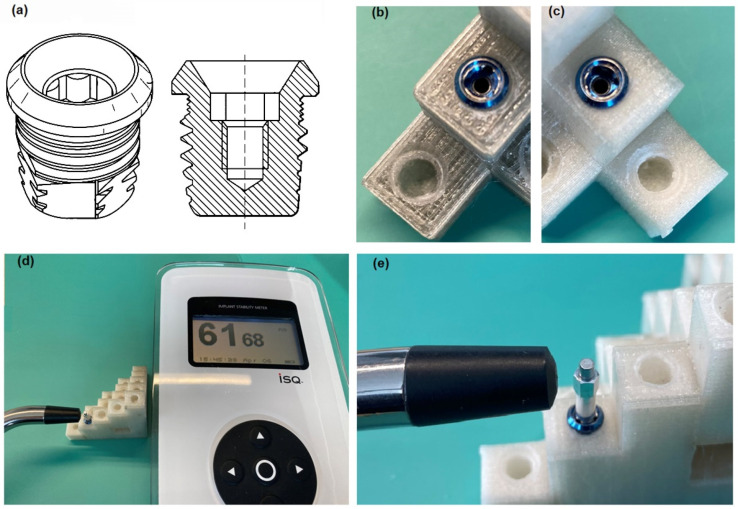
(**a**) Self-tapping VXI300 implant and VXI300 implant cross-section, where a characteristic thread can be seen [11]. (**b**) VXI300 implant mounted in a dry model (700 g PC/5 g PO/3.5 g talc). (**c**) VXI300 Implant mounted in an undried model (700 g PC/5 g PO/3.5 g talc). (**d**) Measurement system: Ostelle ISQ provided by Osstell with a magnetic handpiece and SmertPeg type 30 embedded in Cochlear VI300 implant. (**e**) Magnification: VXI300 implant embedded in test material with SmartPeg type 30 temporarily inserted.

Experimental methods allow determining the final mechanical stability of the inserted implant into the material. For this purpose, non-contact measurements of the stability of the implants inserted into the holes were performed using the Ostelle ISQ device (Ostell, Göteborg, Sweden) and the excited SmatPeg type 30 indicators (Figure 5). During the measurement, the probe is placed perpendicular to the sensor in two places on the circumference of the implant perpendicular to each other, hence the names ‘horizontal’ and ‘vertical’ axes. Implants have different stability in different directions. In most cases, it is possible to measure two resonant frequencies—one high and the other low—which correspond to the directions with the highest and the lowest stability. The Osstell ISQ instrument measures the resonant frequency of the implant in Hz, then translates it into an implant stability quotient (ISQ) in the range of 1 to 100 and displays it on the Osstell ISQ instrument. The higher the ISQ, the greater the implant’s stability [37], and thus also the higher frequency of its vibrations. The increase in frequency reflects the increased stiffness of the bone interface with the implant [38], which, according to the literature, reflects the degree of osseointegration advancement. The resonance frequency analysis (RFA) technique allows measuring the micro-movement of the SmartPeg sensor temporarily tightened to the implant under the influence of lateral load (functional forces that will be applied to the implant) [39].The exact measurement process was performed according to the steps described in the manual ref. 25052-00, prepared by the manufacturer of the Ostell AB measuring tool and ref. E81713 of the manufacturer of the implant VXI300 [40,41]. 

## 3. Results

### 3.1. Qualitative Assessment of Printed Filaments

**Figure 6:**
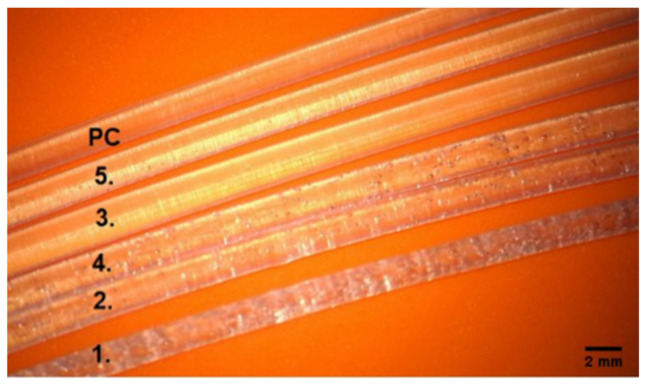
Pictures of the obtained filaments under the Carl ZEISS Vario700 microscope, 19 × magnification. Material in the order counting from the top No. 0-PC, subsequent filament numbers: 1–5, according to the composition from Table 1, filament diameter 1.75 mm.

The printed talc-doped filaments are shown according to the numbering corresponding to their composition described in Table 1 (Figure 6). Changes in clarity were observed with increasing amounts of talc. More heterogeneity—air bubbles appear in filaments 1, 2, 4, where the talc was, respectively, 1.—17.5 g, 2.—7.0 g, 4.—7.0 g. The stability of the modified fiber—the produced filament was 1.75 mm in size and kept the dimension in the range of ±0.05 mm.

### 3.2. Evaluation of the Printout of Calibration Models and the Course of the Drilling Process

The shape of the printed model may have an impact on the obtained results, therefore, a model was selected that allowed us to stabilize it in the tested vice system (drilling, insertion of the implant) and ensure the independence of the examined area and uncomplicated spatial shape. Therefore, choosing a model from connected individual cubes seems to be optimal. Second generation Vistafix implants (dimensions: full implant length 5.35 mm, bone embedded length 4 mm, and diameter 4.475 mm; the diameter of the collar at its widest point 5.2 mm [10]) are implanted in the bones of the skull, most often in the temporal bone, zygomatic bone, frontal bone, maxilla [3,8,10,42]. According to the literature on the anatomy and surgery of laryngological hearing implant implantation, it appears that the thickness of the temporal bone in places of implantation is at least 5.55 ± 1.46 mm on average, and it even reaches 11.15 ± 1.86 mm [43,44,45,46]. Therefore, following the anatomy of the skull and the dimensions of the implant itself, the selected model allows you to freely create a hole for the VXI300 implant (without damaging the side walls of the cube) and the entire surface. Bones fit the implant while leaving a margin of material around the inserted implant, as with real bone.

Since the model’s shape has an uncomplicated structure, the resolution of 3D printing was set at 0.35 mm. This value keeps the surface smooth. In contrast to the commercial model, the edges of the individual layers are noticeable here, which is correct and results from the incremental method used. The individual fibers are imprinted on one another, fusing with each other under the influence of temperature so that the produced pattern becomes a monolith. In the commercial model obtained by the casting method, there are disadvantages, such as air bubbles, that create a perforation of the model inside and outside. The size of the bubbles in the analyzed commercial model reaches a maximum of 1.5 mm.

Printed calibration models, made with the FFF method, differ visually due to the degree of drying of the filaments before 3D printing:

- test model with the composition of 700 g PC/5 g PO/3.5 g talc, cream–white color, non-transparent, visible porosity of the structure (Figure 2a),
- test model with the composition of 700 g PC/5 g PO/3.5 g talc, where the filament was additionally free of water before printing (Figure 2b), gray–transparent color, visible porosity of the structure.

**Figure 7:**
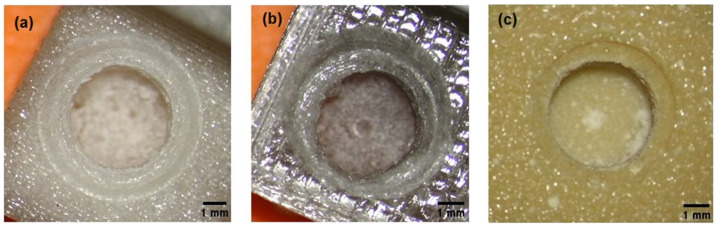
The effects of drilling with surgical drills; from the left, a model from: (**a**) the tested material, (**b**) the tested material after the drying process before its 3D printing, (**c**) a commercial material from Cochlear. X12.5 focal length 225–230 mm.

For comparison, a commercial model: yellow, brittle, made by casting, with visible holes for air bubbles (Figure 7c).

Additionally, it can be seen that the corner specimens at the base of the calibration model No. 11 to 15 had a more compact structure (Figure 2), which translated into an increase in temperature when drilling with drill 1 and drill 2.

The temperature of the material increased during drilling. The mean values of the temperature rise are presented in Table 2. A greater increase in temperature was observed in all the samples for the widening drill (drill 2).

The chips in the tested model (after the drying process) had the shape of springs. Some of the material removed from the hole remained around the hole. The material in the test model with the composition of 700 g PC/5 g PO/3.5 g talc, where the filament was additionally free of water before printing, stuck to the drills to a lesser extent during drilling than the same material without water before 3D printing. On the other hand, the chips in the commercial model were loose, mainly in the form of a powder, and were electrified. In the obtained hole, a distinctive rim could be seen. The effects of the holes made depending on the material used are summarized in Figure 7.

The average temperature rise for the tested materials turned out to be similar. The temperature change during the drilling of holes for all tested materials is presented in Table 2 (Table 2). The highest temperature increase during drilling with drill 2 (average 9.2 °C ± 4.2 °C) was observed for commercial material. Although no differences can be found between drilling temperatures in dry and non-dried material, they are lower than in the commercial model, which is an expected result.

All holes made for the VXI300 implant in the models had the desired shape and diameter size 4.1 ± 0.1 mm for the hole and 5.5 ± 0.1 mm. There were no differences in dimensions between the materials used.

Apart from the 3D print of the model No. 3 from undried filament with a composition of 700 g PC/5 g PO/3.5 g talc, it turned out to be impossible to print models from the remaining undried filaments. The influence of moisture was so unfavorable that, despite many attempts, it was not possible to achieve the required shape and structure of the solid to make the model suitable for further research. Therefore, the prints using the FFF method with the use of the remaining filaments were made when the material was previously dried and removed from moisture. The average temperature increase during the cutting process of the models made of filaments with the tested compositions is shown in Table 3.

When cutting material with drills, characteristic observations of the behavior of the material, including chips, as well as the course of the drilling process itself were described:

- Material no. 1: drilling in the material is easy, the chips are elastic but brittle, they disintegrate and do not remain on the drill; the drill very quickly reaches the set depth, which disturbs the sense of control over it;
- Material no. 2 and 3: optimal drilling, spring-shaped chips—most of them are removed from the drill by themselves, full control during drilling; punched wells have very clear visible boundary lines of the holes;
- Material no. 4: drilling is like rubber, feel resistance on the drill; the shavings stick to the drill, the residual material must be manually removed from the drill; punched wells have very clear visible boundary lines of the holes,
- Material no. 5: no control over drill depth; the drill has resistance, it takes a long time to drill the hole and reach the set depth, the material seems very dense; the chips stick to the drill bit, the drill should be cleaned mechanically; coastlines invisible through melted edges and lumpy bone chips.

In comparison with the above observations, the comparative material—commercial, during cutting with drills was characterized by extremely different features. The shavings were loose dust, which additionally became electrified. It was easy drilling; the drill very quickly, without resistance, formed a hole while cutting; the work of the drill was louder than in the tested material. The widening drill bit felt warm (warm to the touch).

### 3.3. Stability Measurement Results

Commercial model: despite being very fragile, the implants could be inserted completely into the bore with a torque of 40 Ncm.

Model not dried: Hard material, in half of the cases, the implant could not be screwed completely into the tested material with the force of 40 Ncm.

Dry model: All implants were successfully tightened to the end of the prepared holes, a torque of 40 Ncm was sufficient to fully insert the implants. In addition, the drained model has better results that are closer to the commercial model. Perhaps the reduced amount of water in the material during 3D printing allows you to control the printing process better, and thus achieve a material with better parameters.

For all models, the average stability in both directions (vertical–horizontal) was similar or the same—which is the desired effect (Table 4). The closer the stability values in both directions to each other, the better—the more symmetrical dimensions of the prepared hole.

## 4. Discussion 

The conducted tests confirmed that the material obtained during the project implementation is suitable for processing by extrusion and can be used in additive technologies using the FDM method. The obtained layers did not show delamination, and the homogeneity of the surface was consistent and no defragmentation of the edges was observed during the drilling process.

The methods listed above have completely different modeling technology. The commercial model is produced by the casting method, while the tested models based on polycarbonate (PC) doped with talc were made using the FFF (fused filament fabrication) method. The casting method requires a form that will give the desired shape to the model. With the FFF technique, the shape is created based on a digital model created and adapted for this purpose in a graphic program or based on sections obtained from computed tomography examination. Thanks to this, the FFF method gives the advantage of not producing intermediate elements, and the model itself can be freely modified or duplicated before printing. In addition, thanks to the easy archiving (saving the model to a file), you can restore the model after many years in unchanged proportions.

If we are talking about the model’s accuracy, the commercial model depends on the template made most often based on a prosthetic impression. If the impression is made incorrectly or is damaged during storage, the produced model will contain surface errors and will not reproduce the full anatomy. The model printed by the FFF method depends solely on the digital model created based on scans of the elements that we want to reproduce. In the case of 3D printing, the model is reproduced in the same proportions, however, these proportions can be freely modified. The quality of reconstructing the structures will depend on digital filters (e.g., Gauss), which are designed to clean surfaces from digital artifacts, as well as on the resolution of the 3D printer itself and printing parameters. The finer the resolution, the more detailed the print is, with the simultaneous extension of the model production time. The undoubted advantage is that in most printers, resolution can be adjusted, which allows the selection of printing parameters in the best possible way.

Moreover, talc is involved in the formation of more ordered structures, which can be used in combination with PC with an amorphous body structure. In addition, as the amount of talc increases, the values of the yield strength and Young’s longitudinal modulus increase, and the material becomes stronger. Due to the properties of talc—high heat capacity—the stabilizer additionally reduces friction at the interface between talc and PC, dissipates heat, and prevents material tearing at the interface. This is confirmed by the results obtained in the field of cutting (Table 3, Figure 7, and cutting descriptions).

Due to the benefits resulting from 3D printing, such as the personalization of medicine, cost-effectiveness, speed, and ease of production, it is subject to continuous development and research in various fields of medicine [47]. The Vistafix system is perfect as a prosthetic solution in the treatment of extensive craniofacial defects. The additional use of the 3D printing technique for this purpose as a tool for the treatment of diseases related to bone loss [19] seems to be a good direction.

The FDM method is rapid prototyping, but there are still a few limitations in this field: mechanical strength (especially between successive layers), roughness, and shape integrity of the manufactured element [48]. In addition, the effects of FDM or FFF printing may show leakage or deformation of the shape when the printing parameters are not exactly matched to the material used [49].

The proper selection of temperature and speed of extrusion is crucial for the created material: too low a temperature on the screw causes inclusions in the material, causing discontinuity of the granulate fiber in the unplasticized polymer, similarly low pressure may reduce the degree of plasticization [50].

In our research, we have proven that the dried material on a PC matrix with an admixture of talc can be successfully used in FFF printing, provided that the material is dried before the printing process, which is a standard procedure for processing polycarbonate.

When analyzing the results for the samples of the material that were printed in an un-dried form, it turned out that the differences are primarily:

- in appearance and behavior of chips;
- in the ISQ study, the lowest results were observed for the non-dried model, although it was not statistically significant (Table 4).
- in the color and the way of reflecting the light of the printed models (Figure 7), where the PC can be colored in any color because this material can be dyed very easily according to the safety data sheets;

Gomez-Gras et al., in their study, found that the effect of setting the printing parameters does not significantly affect the weight, cost, and production time but has a significant impact on the mechanical and functional properties: strength, stretching, bending, shearing, impact, and fatigue material [24].

The topic of PC reinforcement with inorganic fillers and their influence on the composite obtained in this way is not often discussed in the literature. The purpose of the composite is to improve the properties of the material. Bulanda et al. show that the amount and type of filler significantly influenced the functional properties of composites [51]. On the other hand, the filler reduces the amount of polymer in the material formation process [26].

Material aging-reduced resistance to UV radiation or susceptibility to scratching may affect the life of the manufactured surgical instrument should also be considered.

Thanks to the possibility of the sterilization of the surface of the printed training model, in the future, it will be an opportunity to use it not only in the training field but also in the surgical field; it will enable contact with the tissue, its multiple uses and possible filling of the bone surface necessary in further prosthetic stages.

Therefore, the selection of appropriate processing parameters is of key importance. In this experiment, it was one of the most important stages of the research and involved many repetitions and modifications of the process.

The stability of the implant in the tested materials with a PC matrix according to Table 4 has a lower ISQ value compared to the stability of the implant embedded in the commercial material. However, the results are very similar (on the border of error). The most similar result was obtained for models 3 and 2. In the example of dental implants, in vitro studies show a correlation between RFA values and lateral movements or implant micro-mobility (less lateral movement is associated with higher RFA values), where higher bone density was positively correlated with higher RFA values [39]. This feature has a positive effect on the drilling process, where less noise was observed during cutting in the tested materials compared to the commercial material.

Benefits of the surgical and training evaluation point:

- The material is easy to thermoform, which means that we can freely recreate the angles and planes that interest us;
- The model can be a 1:1 mapping of selected bone fragments of the patient, which will facilitate the planning of the stages of the actual surgery; we obtain a faithful representation of the patient’s anatomy;
- Models made of such a material can be successfully personalized according to the training needs, the surgeon’s skills, or the degree of advancement in surgical training;
- Model renewal (the ability to easily print with exactly the same shapes) does not limit the surgeon to one attempt; it makes it possible to consider many methods of carrying out the procedure and to test them in practice; it also has a didactic value, as the surgeon can test many options, including the risky ones, and on their basis reduce the risk of error;
- There are no contraindications for the model made of the tested material to be sterilized or disinfected, in addition to using various available methods; the use of the material in a sterile field is not excluded.

Benefits from the user comfort assessment point:

- The material looks aesthetic, has no random air bubbles or other undesirable artifacts that could disturb the surgical planning process or affect the overall course of training;
- The material does not raise dust and does not pick up static during cutting;
- The porosity of the material allows you to keep the drill in the correct position, the drill bit does not slip, it is easier to make the first drill and continue the process with the next drill bits (according to the instructions);
- The material did not crumble or chip;
- Quieter operation of the drill in the material;
- Benefits from the economic assessment point:
- Easy availability and relatively low cost of purchasing the components that make up the tested composite;
- An uncomplicated method of creating a composite in the form of a filament for 3D printing (possible to be implemented in non-laboratory conditions);
- The filaments obtained are ready for direct use in commonly available printers (3D printing method: FFF).

## 5. Conclusions

The developed PC material with an admixture of talc is suitable for rapid prototyping using the FFF/FDM method. The condition is that it must first be subjected to a drying process in order to remove moisture.

Among the various compositions of the tested composites, the best in terms of the purpose of use as a training system is the composition No. 2 and No. 3, where the PO is 5.0 g, and talcum is 7.0 g and 3.5 g, respectively.

The printed prototypes allow cutting, which was successfully used in the reconstruction of the surgical procedure—anchoring the BI300 implant. The material does not react with the drill, it does not delaminate nor is the surface of the holes made torn out, and it is possible to screw in and unscrew the implant.

The lack of reflectivity of the tested material allows direct temperature measurement in real time, which in turn allows you to control the cutting process.

The temperature increase itself does not affect the plastic processing of the material—both when making a hole with drill bits made of surgical steel and at higher temperatures.

In addition, the tested material is made of readily available substances, which is not a commercially expensive material, which will undoubtedly affect its versatility and availability in the future. The low cost will positively affect the availability, and thus the operator’s training level and the implantation planning process.

## Figures and Tables

**Table 1:** Composition of the tested filaments.

	The Material Amount [g]		
Filament Number	PC	PO	Talc
1.	700	5.0	17.5
2.	700	5.0	7.0
3.	700	5.0	3.5
4.	700	2.5	7.0
5.	700	2.5	3.5

**Table 2:** The results of the temperature change during cutting for the undried and dried models with the composition of PC–700 g, PO–5.0 g, talc−3.5 g in comparison with the commercial model. The temperature increase (ΔT) for drill 1 and drill 2 separately was calculated as the difference between the highest temperature obtained in the drilling process and the initial temperature of the analyzed surface. Standard divination (SD) was also calculated.

Sample Number	Commercial Model		Model Undryed		Model Dryed	
	ΔT _drill 1_ [°C]	ΔT _drill 2_ [°C]	ΔT _drill 1_ [°C]	ΔT _drill 2_ [°C]	ΔT _drill 1_ [°C]	ΔT _drill 2_ [°C]
1.	1.0	4.2	1.4	5.0	1.4	3.1
2.	1.4	3.3	1.8	5.1	1.7	3.8
3.	0.7	9.0	6.0	4.0	2.9	4.0
4.	1.6	14.6	3.1	4.0	1.8	3.9
5.	1.2	7.7	1.7	3.8	1.5	3.9
6.	0.4	6.4	1.5	4.2	1.8	4.3
7.	0.6	6.8	1.5	5.1	2.9	3.4
8.	0.8	6.4	1.9	4.4	2.5	3.7
9.	1.3	7.9	2.9	3.4	2.2	3.9
10.	1.7	5.1	2.5	3.7	3.0	4.1
11.	2.4	7.0	3.8	5.1	3.6	5.0
12.	2.4	16.5	3.6	5.2	3.4	7.6
13.	6.6	13.5	3.4	7.6	3.7	5.2
14.	6.3	15.1	2.9	4.5	5.9	5.0
15.	6.1	14.4	4.4	14.1	4.4	14.1
**Average**	**2.3**	**9.2**	**2.8**	**5.3**	**2.8**	**5.0**
SD	2.1	4.1	1.2	2.6	1.2	2.7

**Table 3:** The temperature change during drilling of holes for all tested materials. Materials were dried before drilling. A total of 15 wells were made in each material. The difference (ΔT) between the highest temperature obtained in the drilling process and the initial temperature of the analyzed surface was calculated for drill 1 and drill 2. Standard divination (SD) was also calculated.

	Temperature Increase during Cutting with a Drill											
Sample Number	Commercial Model		Model 1 (700 g PC/5.0 g PO/17.5 g Talk)		Model 2 (700 g PC/5.0 g PO/7.0 g Talk)		Model 3 (700 g PC/5.0 g PO/3.5 g Talk)		Model 4 (700 g PC/2.5 g PO/7.0 g Talk)		Model 5 (700 g PC/2.5 g PO/3.5 g Talk)	
	ΔT _drill 1_ [°C]	ΔT _drill 2_ [°C]	ΔT _drill 1_ [°C]	ΔT _drill 2_ [°C]	ΔT _drill 1_ [°C]	ΔT _drill 2_ [°C]	ΔT _drill 1_ [°C]	ΔT _drill 2_ [°C]	ΔT _drill 1_ [°C]	ΔT _drill 2_ [°C]	ΔT _drill 1_ [°C]	ΔT _drill 2_ [°C]
1.	1.0	4.2	0.3	1.9	0.9	2.7	1.4	3.1	0.8	2.6	1.2	1.8
2.	1.4	3.3	1.0	3.3	1.5	4.3	1.7	3.8	1.1	3.3	2.2	5.1
3.	0.7	9.0	1.8	3.9	1.0	7.4	2.9	4.0	1.3	3.1	2.1	6.6
4.	1.6	14.6	1.6	4.2	1.6	4.9	1.8	3.9	1.5	3.9	2.1	4.0
5.	1.2	7.7	1.6	5.2	4.7	8.2	1.5	3.9	1.5	3.0	1.9	4.8
6.	0.4	6.4	3.1	6.9	4.6	8.5	1.8	4.3	0.1	2.6	3.9	5.0
7.	0.6	6.8	3.4	3.6	4.9	6.9	2.9	3.4	1.7	3.0	1.8	4.9
8.	0.8	6.4	3.3	7.2	5.2	6.3	2.5	3.7	1.4	3.1	5.8	7.4
9.	1.3	7.9	3.2	5.2	5.1	10.9	2.2	3.9	1.6	3.6	3.4	5.4
10.	1.7	5.1	2.9	5.4	5.3	8.7	3.0	4.1	1.0	3.3	4.0	5.1
11.	2.4	7.0	3.5	3.9	4.4	9.0	3.6	5.0	2.4	3.5	4.0	5.1
12.	2.4	16.5	1.0	4.6	4.0	8.8	3.4	7.6	2.4	3.7	2.7	4.1
13.	6.6	13.5	3.0	6.2	5.9	5.6	3.7	5.2	1.6	3.9	3.6	5.1
14.	6.3	15.1	3.6	4.4	4.4	8.3	5.9	5.0	1.9	3.8	4.6	5.6
15.	6.1	14.4	3.1	4.8	5.1	8.1	4.4	14.1	2.6	3.7	3.8	5.5
**Average**	**2.3**	**9.2**	**2.4**	**4.7**	**3.9**	**7.2**	**2.8**	**5.0**	**1.5**	**3.3**	**3.1**	**5.0**
SD	2.1	4.2	1.1	1.3	1.7	2.1	1.2	2.6	0.6	0.4	1.2	1.2

**Table 4:** Results of the stability measurement of the VXI300 implant in the tested materials. The implant stability quotient was made for each model tested and for model No. 3 in the undried version. Readings in the horizontal and vertical axis were made for 15 implant anchors in each of the tested models. Stability results were averaged for each material tested.

Implant Stability Quotient							
Material Type	Commercial Model	Model 1 (700 g PC/5 g PO/17.5 g Talc)	Model 2 (700 g PC/5 g PO/7 g Talc)	Model 3 (700 g PC/5 g PO/3.5 g Talc)	Model 4 (700 g PC/2.5 g PO/7 g Talc)	Model 5 (700 g PC/2.5 g PO/3.5 g Talc)	Undried Model 3 (700 g PC/5 g PO/3.5 g Talc)
Vertical axis	70 ± 3	64 ± 2	66 ± 4	67 ± 2	62 ± 3	60 ± 4	59 ± 2
Horizontal axis	71 ± 3	64 ± 2	65 ± 5	67 ± 2	62 ± 3	60 ± 4	59 ± 3

## Data Availability

All the necessary data are contained in the article.

## References

[1] Federspil P., Federspil P.A. (1998). Die epithetische Versorgung von kraniofazialen Defekten. HNO.

[2] Plaza A.M., de Perceval Tara M.P., Fernández A.B.M., Martínez E.B., Ramos M.R., Valadés R.F., López A.E. (2019). Bilateral auricular reconstruction with osseointegrated implant-retained prostheses. Optimization of aesthetic outcomes using virtual planning. J. Stomatol. Oral Maxillofac. Surg..

[3] Visser A., Raghoebar G.M., van Oort R.P., Vissink A. (2008). Fate of implant-retained craniofacial prostheses: Life span and aftercare. Int. J. Oral Maxillofac. Implant..

[4] Brånemark P.I., Hansson B.O., Adell R., Breine U., Lindström J., Hallén O., Ohman A. (1977). Osseointegrated implants in the treatment of the edentulous jaw. Experience from a 10-year period. Scand. J. Plast. Reconstr. Surg. Suppl..

[5] Den Besten C.A., Stalfors J., Wigren S., Blechert J.I., Flymm M., Eeg-Olofsson M., Aggarwal R., Green K., Nelissen R.C., Mylanus E.A. (2016). Stability, survival, and tolerability of an auditory osseointegrated implant for bone conduction hearing: Long-term follow-up of a randomized controlled trial. Otol. Neurotol..

[6] Zarb G.A., Albrektsson T. (1991). Osseointegation—a requiem for the periodontal ligament?. Int. J. Periodontics Restor. Dent..

[7] Chang T.L., Garrett N., Roumanas E., Beumer J. (2005). Treatment satisfaction with facial prostheses. J. Prosthet. Dent..

[8] Federspil P.A. (2009). Implant-retained craniofacial prostheses for facial defects. GMS Curr. Top. Otorhinolaryngol. Head Neck Surg.

[9] Granström G. (2007). Craniofacial osseointegration. Oral Dis..

[10] (2012). Cochlear Bone Anchored Solutions AB. Cochlear Vistafix 3 System. Key Dimensions and Material Information. https://www.cochlear.com/f3fdc71c-07a1-4c44-99b0-c3e4b8dfeff5/E82458A_Cochlear_Baha_BIA400_Data_Sheet_GB.pdf?MOD=AJPERES&CONVERT_TO=url&CACHEID=f3fdc71c-07a1-4c44-99b0-c3e4b8dfeff5.

[11] Jinton L., Holgersson E., Elmberg P. (2015). Bone Anchored Fixture for a Medical Prosthesis. U.S. Patent.

[12] (2012). Cochlear Bone Anchored Solutions AB. Treatment and Surgery Guide. Cochlear™ Vistafix® 3 System A Bone Anchored Prosthetic Solution. https://www.cochlear.com/29e502a1-c752-44b8-97bd-5a376c197209/VFX001+Iss1+MAY12+Vistafix+Surgery+Guide.pdf?MOD=AJPERES&CACHEID=29e502a1-c752-44b8-97bd-5a376c197209.

[13] Chrcanovic B.R., Nilsson J., Thor A. (2016). Survival and complications of implants to support craniofacial prosthesis: A systematic review. J. Craniomaxillofac. Surg..

[14] Tolman D.E., Taylor P.F. (1996). Bone-anchored craniofacial prosthesis study: Irradiated patients. Int. J. Oral Maxillofac. Implant..

[15] Subramaniam S.S., Breik O., Cadd B., Peart G., Wiesenfeld D., Heggie A., Gibbons S.D., Nastri A. (2018). Long-term outcomes of craniofacial implants for the restoration of facial defects. Int. J. Oral. Maxillofac. Surg..

[16] Vijverberg M.A., Verhamme L., van de Pol P., Kunst H.P.M., Mylanus E.A.M., Hol M.K.S. (2019). Auricular prostheses attached to osseointegrated implants: Multidisciplinary work-up and clinical evaluation. Eur. Arch. Otorhinolaryngol..

[17] Huang J.J., Ren J.A., Wang G.F., Li Z.A., Wu X.W., Ren H.J., Liu S. (2017). 3D-printed “fistula stent” designed for management of enterocutaneous fistula: An advanced strategy. World J. Gastroenterol..

[18] Tappa K., Jammalamadaka U. (2018). Novel Biomaterials Used in Medical 3D Printing Techniques. J. Funct. Biomater..

[19] Posa F., Di Benedetto A., Ravagnan R., Cavalcanti-Adam E.A., Muzio L.L., Percoco G., Mori G. (2020). Bioengineering Bone Tissue with 3D Printed Scaffolds in the Presence of Oligostilbenes. Materials.

[20] Alaboodi A.S., Sivasankaran S. (2018). Experimental design and investigation on the mechanical behavior of novel 3D printed biocompatibility polycarbonate sca_olds for medical applications. J. Manuf. Process..

[21] Flak T., Trejnowska E., Skoczyński S., Gabor J., Swinarew B., Grzywnowicz K., Okła H., Jasik K., Stanula A., Brożek G. (2021). Novel Antibacterial Modification of Polycarbonate for Increment Prototyping in Medicine. Materials.

[22] Covestro Macrolon® 2600 Polycarbonate Datasheet. http://www.lookpolymers.com/pdf/Covestro-Makrolon-2600-Polycarbonate.pdf.

[23] Peterson J., Dechow P.C. (2003). Material properties of the human cranial vault and zygoma. Anat. Rec. Part A Discov. Mol. Cell. Evol. Biol..

[24] Gomez-Gras G., Abad M.D., Pérez M.A. (2021). Mechanical performance of 3D-printed biocompatible polycarbonate for biomechanical applications. Polymers.

[25] West M., Ruys A., Bosi S. The Effect of the Ultraviolet Radiation Environment of LEO upon Polycarbonate Materials. Proceedings of the 43rd AIAA Aerospace Sciences Meeting and Exhibit-Meeting Papers.

[26] Swinarew A.S., Swinarew B., Flak T., Okła H., Lenartowicz-Klik M., Barylski A., Popczyk M., Gabor J., Stanula A. (2021). The Evaluation of Simulated Environmental Degradation of Polycarbonate Filled with Inorganic and Organic Reinforcements. Polymers.

[27] Plastics Base PC Polycarbonate. https://www.tworzywa.pl/wiedzopedia/baza-tworzyw/81,poliweglan-pc,polimer.html.

[28] Identification of plastics. https://docplayer.pl/10653085-Identyfikacja-tworzyw-sztucznych.html.

[29] Banasiak A., Sterzyński T. (2002). Structure and properties of PE + Talc polymeric composites. Composites.

[30] Rokpol D2002 Datasheet PCC Rokita. https://www.products.pcc.eu/pl/id/8980/rokopol-d2002/.

[31] Wodarski K. Expertise 2.2. Analysis of the development of key technologies in the field of metallic, ceramic, polymer and composite products. http://roz4.polsl.pl/wp-content/uploads/2019/11/E-4_2.2_Analiza-rozwoju-kluczowych-technologii.pdf.

[32] Swinarew A., Grobelny Z., Jasik K., Rozwadowska B., Nowicki G., Flak T., Gabor J., Łężniak M., Okła H. (2015). Sposób otrzymywania modyfikowanych poliestrów, zwłaszcza na bazie poliwęglanu, polilaktydu, lub modyfikowanych kopolimerów i modyfikowane poliestry lub modyfikowane kopolimery otrzymane tym sposobem. Patent.

[33] Swinarew A., Grobelny Z., Jasik K., Rozwadowska B., Nowicki G., Flak T., Gabor J., Łężniak M., Okła H. (2015). Sposób wytwarzania poliuretanu modyfikowanego nanokrzemionką. Polymertech spółka z ograniczoną odpowiedzialnością. Patent.

[34] MCroucher (2012). 5 mm Calibration Cube Steps. https://www.thingiverse.com/thing:24238.

[35] Björn G., Frimanson J. (2017). Mastoid Bone Start Drill Bit. U.S. Patent.

[36] Nelissen R.C., den Besten C.A., Faber H.T., Dun C.A., Mylanus E.A., Hol M.K. (2016). Loading of osseointegrated implants for bone conduction hearing at 3 weeks: 3-year stability, survival, and tolerability. Eur. Arch. Otorhinolaryngol..

[37] Balleri P., Cozzolino A., Ghelli L., Momicchioli G., Varriale A. (2002). Stability measurements of osseointegrated implants using Osstell in partially edentulous jaws after 1 year of loading: A pilot study. Clin. Implant. Dent. Relat. Res..

[38] Meredith N., Alleyne D., Cawley P. (1996). Quantitative determination of the stability of the implant-tissue interface using resonance frequency analysis. Clin. Oral Implant. Res..

[39] Schallhorn R.A. (2017). Resonance Frequency Analysis in Implant Dentistry. Decis. Dent..

[40] Ostell A.G. Osstell ISQ Quick Guide. Guidelines for Measuring Implant Stability using Osstell ISQ on Cochlear™ Baha® Implants and Abutments. https://manuals.plus/m/24c388e5f0baa1d4c0f140fbb718c8c7bfd6275d5be247abe6163c06a627a3ed.

[41] Nelissen R.C., Stalfors J., de Wolf M.J., Flynn M.C., Wigren S., Eeg-Olofsson M., Green K., Rothera M.P., Mylanus E.A., Hol M.K. (2014). Long-term stability, survival, and tolerability of a novel osseointegrated implant for bone conduction hearing: 3-year data from a multicenter, randomized, controlled, clinical investigation. Otol Neurotol..

[42] Charpiot A., Chambres O., Herve J.F., Million P., Riedinger A.M., Hemar P. (2006). Osteointegrated cranio-facial implants: 49 patients report. Rev. Laryngol. Otol. Rhinol..

[43] Gawęcki W., Gibasiewicz R., Marszał J., Błaszczyk M., Gawłowska M., Wierzbicka M. (2022). The evaluation of a surgery and the short-term benefits of a new active bone conduction hearing implant—the Osia®. BJORL. Braz. J. Otorhinolaryngol..

[44] Marszal J., Gibasiewicz R., Blaszczyk M., Gawlowska M.B., Gawecki W. (2021). Piezoelectric bone conduction hearing implant Osia®—audiological and quality of life benefits. Otolaryngol. Pol..

[45] Rahne T., Svensson S., Lagerkvist H., Holmberg M., Plontke S.K., Wenzel C. (2021). Assessment of Temporal Bone Thickness for Implantation of a New Active Bone-Conduction Transducer. Otol. Neurotol..

[46] Ekşi M.S., Güdük M., Usseli M.I. (2021). Frontal Bone is Thicker in Women and Frontal Sinus is Larger in Men: A Morphometric Analysis. J. Craniofac. Surg..

[47] Ventola C.L. (2014). Medical Applications for 3D Printing: Current and Projected Uses. Pharm. Ther..

[48] Sharafeldin M., Jones A., Rusling J.F. (2018). 3D-Printed Biosensor Arrays for Medical Diagnostics. Micromachines.

[49] Wang X., Jiang M., Zhou Z., Gou J., Hui D. (2017). 3D printing of polymer matrix composites: A review and prospective. Compos. Part B Eng..

[50] Haryńska A., Kucinska-Lipka J., Sulowska A., Gubanska I., Kostrzewa M., Janik H. (2019). Medical-Grade PCL Based Polyurethane System for FDM 3D Printing—Characterization and Fabrication. Materials.

[51] Bulanda K., Oleksy M., Oliwa R., Budzik G., Przeszłowski Ł., Fal J., Jesionowski T. (2021). Polymer Composites Based on Polycarbonate (PC) Applied to Additive Manufacturing Using Melted and Extruded Manufacturing (MEM) Technology. Polymers.

